# Prevalence of temporomandibular disorders diagnosis in patients treated with Herbst appliance: a systematic review and meta-analysis

**DOI:** 10.1186/s12903-023-03738-w

**Published:** 2024-01-28

**Authors:** Giuseppe Minervini, Marco Di Blasio, Rocco Franco, Maria Maddalena Marrapodi, Benedetta Vaienti, Marco Cicciù, Vincenzo Ronsivalle

**Affiliations:** 1 Saveetha Dental College and Hospitals, Saveetha Institute of Medical and Technical Sciences (SIMATS), Saveetha University, Chennai, Tamil Nadu India; 2 Multidisciplinary Department of Medical-Surgical and Dental Specialties, University of Campania Luigi Vanvitelli, Caserta, 81100 Italy; 3 Department of Medicine and Surgery, University Center of Dentistry, University of Parma, Via Gramsci 14, 43126 Parma, Italy; 4 Department of Biomedicine and Prevention, University of Rome “Tor Vergata”, 00100 Rome, Italy; 5 Department of Woman, Child and General and Specialist Surgery, University of Campania “Luigi Vanvitelli”, 80121 Naples, Italy; 6 Department of Biomedical and Surgical and Biomedical Sciences, Catania University, 95123 Catania, Italy

**Keywords:** Herbst, TMD, Class II treatment

## Abstract

**Background:**

The Herbst appliance is an excellent therapy for treating class II malocclusions with increased overjet. Its mechanics involve propelling the mandibular bone using two pistons the patient cannot remove. The so-called bite-jumping keeps the mandible in a more anterior position for a variable period, usually at least 6 months. This appliance does not inhibit joint functions and movements, although there are scientific papers in the literature investigating whether this appliance can lead to temporomandibular disorders. This systematic review aims to evaluate whether Herbst’s device can cause temporomandibular diseases by assessing the presence of TMD in patients before and after treatment.

**Methods:**

A literature search up to 3 May 2023 was carried out on three online databases: PubMed, Scopus and Web of Science. Only studies that evaluated patients with Helkimo scores and Manual functional analysis were considered, as studies that assessed the difference in TMD before and after Herbst therapy. Review Manager version 5.2.8 (Cochrane Collaboration) was used for the pooled analysis. We measured the odds ratio (OR) between the two groups (pre and post-Herbst).

**Results:**

The included papers in this review were 60. Fifty-seven were excluded. In addition, a manual search was performed. After the search phase, four articles were considered in the study, one of which was found through a manual search. The overall effect showed that there was no difference in TMD prevalence between pre-Herbst and post-Herbst therapy (OR 0.74; 95% CI: 0.33–1.68).

**Conclusion:**

Herbst appliance seems not to lead to an increase in the incidence of TMD in treated patients; on the contrary, it appears to decrease it. Further studies are needed to assess the possible influence of Herbst on TMDs.

## Introduction

The Herbst appliance is a commonly used orthodontic appliance for treating skeletal Class II malocclusions, which involves the mandible being positioned too far behind the maxilla. Herbst appliance keeps the mandible in a more anterior position. The device is attached to the upper and lower teeth and is designed to advance the mandible forward, thereby correcting the malocclusion. However, there have been reports of temporomandibular disorders (TMD) associated with using the Herbst appliance [1, 2].

TMD is a collective term used to describe a range of clinical problems that involve the temporomandibular joint (TMJ) and associated muscles. Temporomandibular disorders encompass a range of clinical conditions affecting the temporomandibular joint, masticatory muscles, and surrounding tissues. TMDs can lead to pain, dysfunction, and a diminished quality of life for affected individuals. This article aims to comprehensively review the current state of knowledge regarding TMDs, including their etiology, clinical manifestations, diagnosis, and management.

TMDs have a multifactorial etiology, often involving a combination of biological, psychosocial, and environmental factors. Contributing factors include trauma, malocclusion, parafunctional habits, genetics, and psychological stress. A thorough understanding of these factors is essential for both prevention and effective management of TMDs.

The clinical manifestations of TMDs vary widely and may include pain, joint noises, limited mouth opening, muscle tenderness, and headaches. Patients may also experience associated symptoms such as ear pain, dizziness, and difficulty chewing. A precise and detailed clinical evaluation is crucial for accurate diagnosis.

These disorders can cause pain, discomfort, and dysfunction in the jaw joint, limiting the ability to speak, chew, and even breathe. TMD can result from various factors, including trauma, arthritis, muscle tension, or abnormal occlusion [3]. Opinions on the significance of occlusion range from claiming that it is the primary causative element and saying that there is no association at all. While some writers argue that occlusion has no part in the development of TMD symptoms, others contend that aetiological variables are more closely related to behavioral, psychological, and neurological issues. Furthermore, there is no proof that individuals with malocclusion have a higher prevalence of TMD, but the connection between the two conditions is still debatable. Studies have not consistently demonstrated evidence to support the theories regarding the occlusal relationships, which include working side interferences, non-working side interferences, and the discrepancy between the centric occlusion (CO) and centric relation (CR), as contributing factors to TMD.

There are no discernible variations between posterior interferences, non-functioning contacts, and a lateral slide from CR to CO that correspond with TMD symptoms. This seems to be due to the complex character of the different indications and symptoms as well as the absence of relevant and trustworthy techniques for assessing the blockage.

The use of orthodontic appliances, such as the Herbst appliance, has been implicated in the development of TMD. This may be due to the altered biomechanics of the jaw joint caused by the device, which can lead to joint inflammation, muscle strain, and instability [4–6]. The Herbst appliance can also cause an increase in muscle activity, which can result in muscle fatigue and pain, causes this due to an unnatural forced mandibular position.

There is limited research on the association between the Herbst appliance and TMD, and the evidence is conflicting. Some studies have reported an increased incidence of TMD in patients treated with the Herbst appliance, while others have found no association. However, it is generally agreed that patients treated with the Herbst appliance should be closely monitored for the development of TMD symptoms [7–18]. Some studies with Herbst’s application have seen a lack of TMD symptoms, or a similar incidence rate to the rest of the population. While others have shown an amplification of TMD symptoms, both spontaneous and on palpation.

In conclusion, while the Herbst appliance is an effective orthodontic treatment for skeletal Class II malocclusions, it may be associated with the development of TMD. Further research is needed to clarify this association’s nature and identify strategies to prevent or minimize the risk of TMD in patients undergoing Herbst treatment [19–23].

Additionally, it is important for orthodontists to carefully assess the patient’s TMJ health before recommending the Herbst appliance and to monitor the patient closely throughout the treatment process. Regular follow-up appointments should be scheduled to detect and manage any potential TMD symptoms [24].

The diagnosis of TMD can be challenging, as the symptoms can vary widely, and there is no definitive test for the condition. Symptoms can include jaw pain, clicking or popping noises in the joint, difficulty opening or closing the mouth, headaches, and ear pain. Treatment for TMD can include lifestyle changes, such as stress reduction and jaw exercises, as well as medications, physical therapy, and in severe cases, surgery [25].

Overall, while the Herbst appliance is a valuable orthodontic treatment option for patients with skeletal Class II malocclusions, it is essential to be aware of the potential risk of TMD associated with its use. By carefully evaluating patients for TMJ health and monitoring them closely during treatment, orthodontists can minimize the risk of TMD and ensure the best possible outcomes for their patients [26, 27].

This systematic review aims to evaluate the occurrence of temporomandibular disorders in patients undergoing orthodontic treatment with Herbst’s appliance.

## Materials and methods

### Search strategy

We looked through Scopus, Web of Science, and PubMed for publications published between the beginning and May 1, 2023. The search method used to look up documents in search engines is shown in Table 1.

**Table 1:** Search strategy

***PubMed*** **(temporomandibular disorders OR orofacial pain) AND herbst** (“temporomandibular joint disorders”[MeSH Terms] OR (“temporomandibular”[All Fields] AND “joint”[All Fields] AND “disorders”[All Fields]) OR “temporomandibular joint disorders”[All Fields] OR (“temporomandibular”[All Fields] AND “disorders”[All Fields]) OR “temporomandibular disorders”[All Fields] OR (“facial pain”[MeSH Terms] OR (“facial”[All Fields] AND “pain”[All Fields]) OR “facial pain”[All Fields] OR (“orofacial”[All Fields] AND “pain”[All Fields]) OR “orofacial pain”[All Fields])) AND “herbst”[All Fields]	
***Web of Science*** (temporomandibular disorders OR orofacial pain) AND Herbst (ALL FIELDS) ***Scopus***	
TITLE (temporomandibular disorders OR orofacial pain) AND Herbst	

Additionally, we manually looked through published topical and systematic reviews on related subjects.

The Cochrane Handbook for Systematic Reviews of Interventions and the Preferred Reporting Items for Systematic Reviews (PRISMA) guidelines 2020 served as our guides when conducting this systematic review. The protocol for the systematic review has been registered as CRD42022354011 on the International Prospective Register of Systematic Reviews (PROSPERO).

### Eligibility criteria

We applied the following Population, Intervention, Comparator, and Outcomes (PICO) model to assess the document eligibility:

P) Participants consisted of Class II division 1 patient.

I) The Intervention consisted of orthodontic treatment with the Herbst appliance.

C) The Comparison consisted of a clinical analysis for temporomandibular disorders before and after treatment with Herbst’s appliance in the same patients.

O) The Outcome was the occurrence of temporomandibular disorders in orthodontic patients treated with Herbst applications.

Only studies that provided prevalence data before and after treatment were included. We established the following exclusion criteria: 1) diagnosis of rheumatic diseases or chronic inflammatory disorders (e.g., rheumatoid arthritis, juvenile arthritis, idiopathic arthritis, psoriatic arthritis); 2) diagnosis of fibromyalgia; 3) congenital abnormalities or neoplastic conditions in the TMJ region; 4) studies that included subjects who underwent arthrocentesis or intra-articular infiltration; 5) cross-over study design; 6) language other than English (we considered only articles written in English); 7) unavailability of full-text (e.g., posters and conference abstracts); 8) studies involving animals; 9) review articles (topical or systematic); 10) case reports/series; 11) evaluation of TMD prior Herbst therapy.

### Data extraction

The data extracted were collected in a digital database.

The following data were extracted by two reviewers (MDB and BV). 1) First author; 2) Year of publication; 3) Nationality; 4) Number of study participants; 5) Age of study participants; 6) Diagnostic criteria/tools used for the diagnosis of TMD; 7) Prevalence in patients before treatment; 8) Prevalence in patients post-treatment.

### Quality assessment

The risk of bias in the included studies was evaluated by two reviewers (MC and GM) using the Cochrane risk-of-bias instrument for randomized trials (RoB 2), Version 2. An established method for assessing the caliber of randomized trials is the Cochrane RoB 2 instrument. Random sequence generation, allocation concealment, participant and staff blinding, outcome assessment blinding, inadequate outcome data, and selective reporting are the six potential bias domains that are taken into account. Any differences of opinion were discussed with a third reviewer (MDB) until a consensus was obtained.

### Statistical analysis

The software Review Manager version 5.2.8 (Cochrane Collaboration, Copenhagen, Denmark; 2014) was used to perform the pooled analysis. We measured the Odds Ratio (OR) between the two groups (prevalence of TMD in pre-Herbst and post-Herbst). The Higgins Index (*I*^*2*^) and the chi-square test were implemented to assess Heterogeneity among studies. We classified heterogeneity as follows: low heterogeneity (< 30%), medium heterogeneity (30–60%), and high heterogeneity (> 60%).

## Results

### Study characteristics

**Fig. 1:**
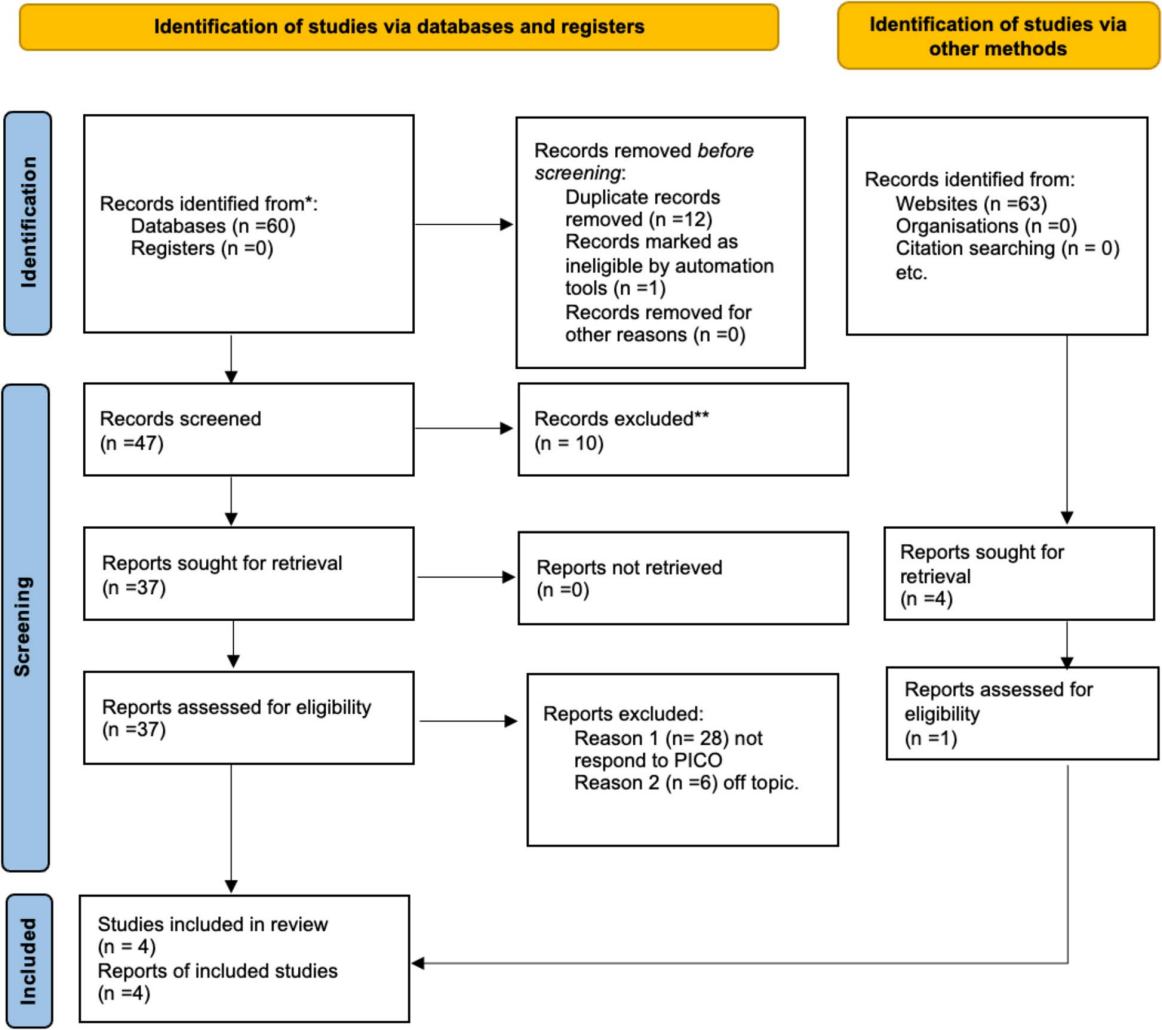
Prisma flowchart. *Page MJ, McKenzie JE. The PRISMA 2020 statement: an updated guideline for reporting systematic reviews. BMJ. 2021 May 11,2023*

Three studies were included in the systematic review and were considered for the metanalysis, as illustrated in the PRISMA 2020 flowchart in Fig. 1. Sixty articles were selected after the search. One report was excluded before the screening because it was not in English. Twelve records were duplicates and, therefore, were excluded. The remaining 47 articles were selected for the title and abstract screening to evaluate whether they met the PICO criteria. Among these, ten studies were excluded because they were reviews, clinical cases, book chapters etc. The remaining 37 articles were selected, and full-text screening was performed. Therefore, 34 pieces were eliminated, as 28 did not meet PICO, and six were off-topic. Therefore three articles were considered by an electronic search. In addition, a manual search of the bibliography and major sites was performed, and an additional article was included. In the end, four papers were included in the study. The included studies have been published between 1982 and 2019. The studies are longitudinal study. All these studies considered the presence of TMD before and after Herbst therapy. They all assessed symptoms with the Helkimo index and Manual Functional analysis. Helkimo was credited as being a pioneer in the development of an index to gauge the level of pain and severity in TMD sufferers. Registration of subjective symptoms applying for the Helkimo Index required a questionnaire-based survey. Questionnaire comprised two parts: Anamnestic component which includes answers to questions in “yes” or “no”.Clinical analysis evaluate the pain and the mouth opening. The two data are combined and based on responses and symptoms a grade from 0 no symptoms to 2 disabling symptoms. Three additional categories were added to Helkimo’s index: occlusal dysfunction, clinical, and anamnesis.Manual Functional Examination assessed patients’ pain and difficulty in opening by categorizing them from 0 to 2. The data extracted from each study, as reported in the paragraph “data extraction,” were written in Table 2.

**Table 2:** Principal elements of the studies which formed part of the present systematic analysis

Author	Year	Nationality	Number of study participants	Age of study participants	Diagnostic criteria	Prevalence of TMD before treatments	Prevalence of TMD after treatments
Pancherz	1982	Sweden	20 patients	11–14 yrs	Helkimo index	Symptomatic: 20% Asymptomatic: 80%	Symptomatic: 45% Asymptomatic: 55%
Aidar	2013	Brazil	32 patients	12.8 yrs	Hellkimo index	Asymptomatic: 18,75% Mild dysfunction: 75% Severe: 6,25%	Asymptomatic: 18,75% Mild: 78,15% Severe: 3,12%
Ruf and Bock	2019	Germany	72 patients	13.4 yrs	Helkimo index RDC/TMD	Symptomatic: 38.5% Asymptomatic: 61,5%	Symptomatic: 27,3% Asymptomatic: 72,7%
Ruf	1999	Germany	62 patients	RDC/TMD	Manual Functional analysis (MFA)	Symptomatic: 6,45% Asymptomatic: 93,55%	Symptomatic: 14,51% Asymptomatic: 85,49%

### Main findings

The included subjects in this review were 147 (71 females and 76 males, mean age 13,3). For each patient, the presence of TMD was assessed at least immediately before treatment with Herbst and immediately after the end of therapy; in the case of studies 1 and 4, the last follow-up is up to 12 months; in the case of study 3 it is greater than or equal to 15 years. A population of 186 patients in whom the onset of symptoms was assessed either by the Helkimo score or by Manual functional analysis was taken for the meta-analysis.

Demographics and clinical data of the involved subjects are presented in Table 2.

In the study by Pancherz and Anehus-Pancherz, 20 male patients were enrolled and successfully treated for six months. The evaluation of the temporomandibular joint included a questionnaire and a clinical examination. The anamnestic part had questions regarding sounds and pain from the TMJ, pain in the masticatory muscles, and biting and chewing difficulties. The answer could be yes or no. The clinical evaluation included the range of mandibular movements (maximum opening capacity, maximum lateral movements and maximum protrusion) according to Ingervall (1970), the presence or absence of joint noises during mandibular movements and tenderness on palpation using the method of Carlsson and Helkimo (1972) of TMJ and masticatory muscle. The questionnaire results indicated that joint sounds (clicking) were noticed by two patients before treatment, but the clicking had disappeared; furthermore, no patients reported pain in the masticatory muscles or TMJ during treatment. On clinical examination, the maximum and maximum protrusive openings remained unchanged before and after treatment; only the lateral movements showed a slight decrease (1.9 mm on average) during treatment, returning to the initial values after treatment. No joint noise was detected twelve months after the end of treatment (although two patients were positive for this sign before treatment). Tenderness on palpation at the TMJ was detected in four patients before treatment, only in two twelve months after treatment. Tenderness on palpation of one or more masticatory muscles was seen in five patients before and in two twelve months after treatment (mainly the posterior belly of the digastric muscle followed by the lateral pterygoid muscle) [28].

In the study conducted by Aidar et al., patients were clinically examined to determine the Helkimo CDI at three-time intervals: immediately before Herbst treatment (T1), at the end of Herbst.

treatment (T3) and the end of phase 2 orthodontic treatment (T4). There were no changes in 26 (86.6%), 16 (76.2%) and 16 (76.2%) patients at T1-T3 (*p* = 1.000), T3-T4 (*p* = 1.000) and T1-T4 (*p* = 1.000), respectively. There were changes in 4 (13.3%), 5 (23.8%) and 5 (23.8%) patients at T1-T3, T3-T4 and T1-T4, respectively [29].

In Ruf and Bock’s work, 33 patients were analysed at three-time points: before treatment (T0), after treatment (T1) and at an average follow-up of 19.2 years (T2). The patients were evaluated based on the Helkimo index and with the research diagnostic criteria for temporomandibular disorders (RDC/TMD) and diagnostic criteria for temporomandibular disorders (DC/TMD). At each of these three points, most patients (82–88%) never presented anamnestic symptoms of TMD: an improvement trend between T0 and T1 and a recurrence trend between T1 and T2 was noted, however, without statistical significance. Clinical dysfunction (Helkimo index D) was never shown between 55 and 73% of the patients at the three-time points. An improvement between T0 and T1 and a recurrence between T1 and T2 can also be seen here, but without statistical significance [30].

In the study by Ruf et al., 62 patients (35 females and 27 males) were analyzed, with a mean age at the start of treatment of 14.4 years. The patients were clinically examined according to Manual Functional Analysis (MFA) at three time points: before Herbst treatment (T1), immediately after Herbst removal (T2), and one year after Herbst removal (T3). At T1, capsulitis of the low stratum was detected in 21 patients (5 symptomatic and 16 sub-symptomatic); at T3, only five patients. Clinical signs of disc displacement were detected at T1 in 9 patients (11 joints): in two joints, a partial disc displacement with reduction (PDDwR); in five joints, a total disc displacement with reduction (TDDwR); and in four joints, a full disc displacement without reduction (TDDnoR). At T2, only one joint showed clinical signs of disc displacement, and at T3, two affected joints (1 patient) with a TDDwR were identified. Crepitus was detected in two patients (number 2 and 39) at both joints at T1, in one joint (patient number 2) at T2 and in three joints (patients number 3 and 39) at T3 [31].

### Meta-analysis

The included studies had medium heterogeneity (*I*2 = 56%). Therefore, the meta-analysis was conducted by random model effect. We considered as an outcome the TMD prevalence pre-Herbst and post-Herbst. During the meta-analysis, we took the Helkimo index as the diagnostic criterion of TMD for the three studies and one MFA that divided the symptomatology into three grades, absence, moderate and high. Therefore we considered the patients assessed by Helkimo score as 0 unaffected, and grade 2 and 3 combined and united them as one group of TMD patients. We did the same for the study with MFA, combining the moderate and high group as the affected group. The parameters between these two indexes are similar, so we combined their effects for the meta-analysis. In addition, given the marginal risk of bias anyway, we used random effects. Therefore, to make the results homogeneous, we combined the medium and high groups and unified them into a single group representing TMD patients.

**Fig. 2:**
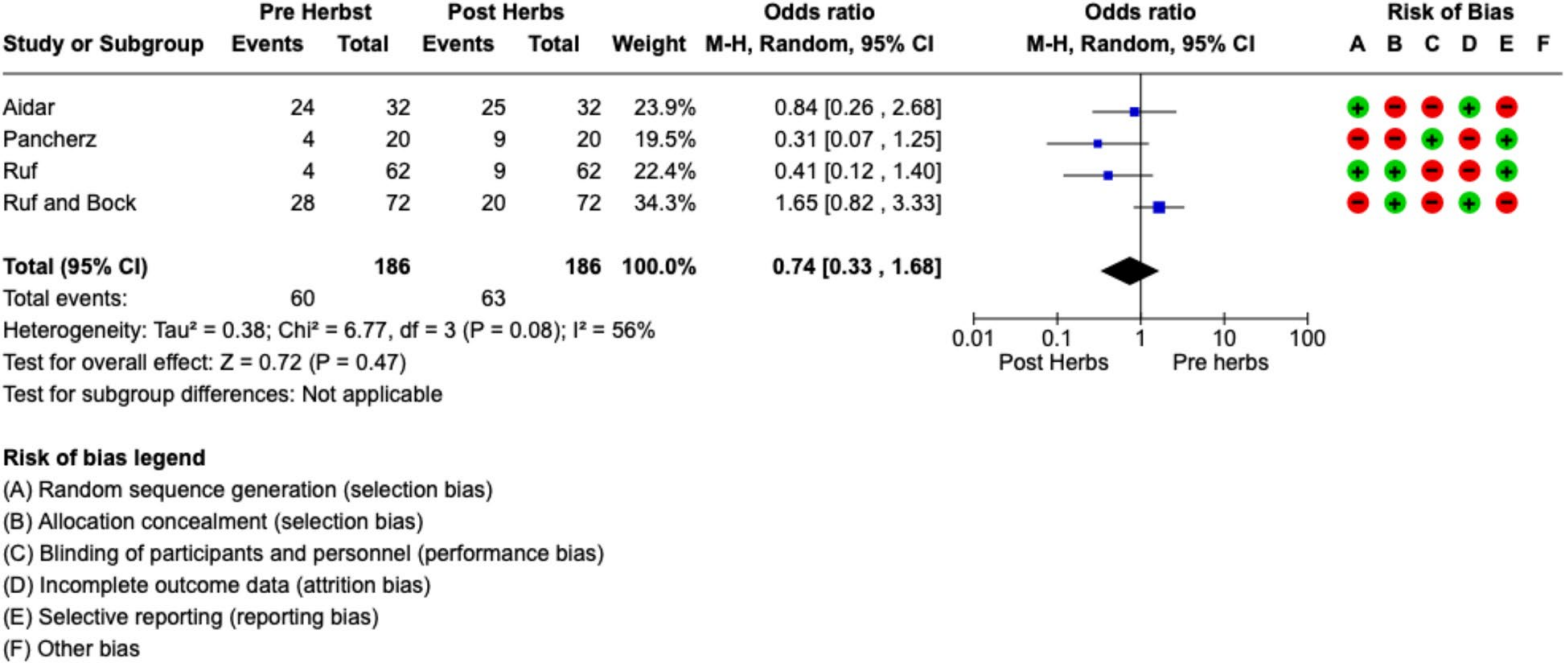
Forest plot of the meta-analysis

The overall effect, reported in the forest plot (Fig. 2), showed that there was no difference in TMD prevalence between pre-Herbst and post-Herbst therapy (OR 0.74; 95% CI: 0.33–1.68), suggesting that Herbst is not a factor that can cause or at least exacerbate the symptomatology of TMD.

### Quality assessment and risk of bias

**Fig. 3:**
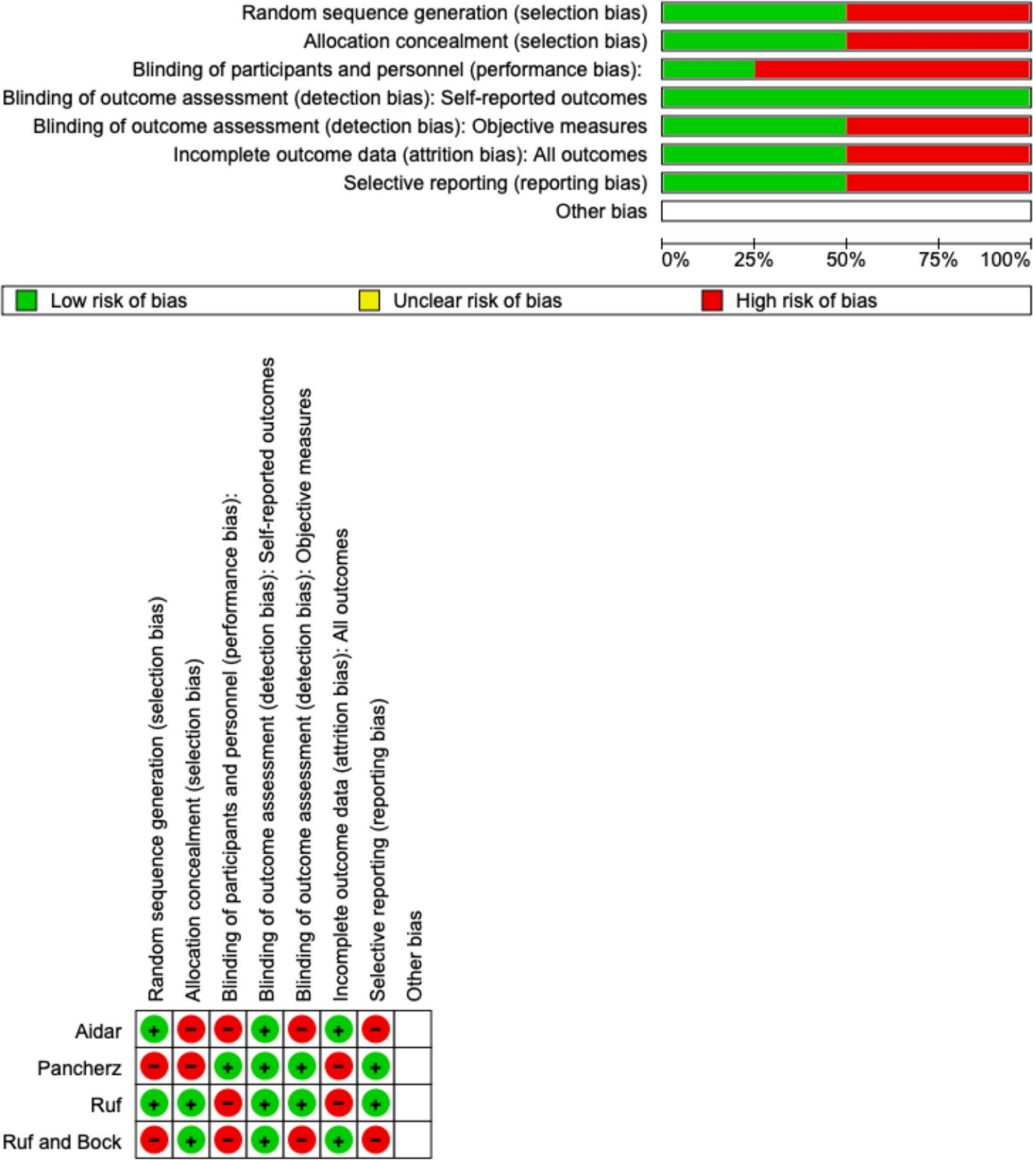
Risk of bias of the included studies

The risk of bias in the included studies was reported in Fig. 3. Regarding the randomization process and allocation concealment, two studies ensured a high risk of bias. Only one study excluded a performance; all the studies confirmed an increased risk of detection bias (self-reported outcome), and 2 of the included studies present low detection bias (objective measures) (Fig. 3). Two studies ensure a low risk regarding attrition and reporting bias.

## Discussion

The Herbst appliance is a popular orthodontic device used to treat mandibular deficiencies by advancing the mandible. While the Herbst appliance can effectively correct mandibular retrognathism, it cause influence temporomandibular disorders (TMDs) in some patients. TMDs are a group of diseases that affect the temporomandibular joint (TMJ) and the muscles responsible for jaw movement [32–37].

Evidence suggests that the Herbst appliance can cause TMDs in some patients. Some studies found that patients who had undergone Herbst treatment experienced TMD symptoms [38, 39]. These symptoms included pain in the TMJ, muscle pain, and limited jaw movement. The study also found that patients with pre-existing TMD symptoms were more likely to experience a worsening of their symptoms after Herbst treatment [40, 41].

The mechanism, according to studies in the literature by which it causes TMD is due to a stretching of the TMJ resulting in muscle tension and stretching of the disc [42, 43]. However, the device is believed to cause increased stress on the TMJ and the muscles responsible for jaw movement. This increased stress can lead to inflammation, muscle spasms, and pain [44–46]. While various treatment modalities exist, the use of Botulinum Toxins (BoNT) has emerged as a promising therapeutic option. Botulinum Toxins, produced by the bacterium *Clostridium botulinum*, are potent neurotoxins that act by inhibiting the release of acetylcholine at the neuromuscular junction. This leads to temporary muscle paralysis, making BoNT an attractive option for conditions characterized by hyperactivity of muscles, such as those involved in mastication. BoNT’s muscle-paralyzing effects have been leveraged to alleviate muscle hyperactivity and spasm in TMD patients, providing relief from pain and improving jaw function [47].

BoNT has demonstrated analgesic effects beyond its muscle-relaxing properties, suggesting a potential role in managing the pain associated with TMDs. The mechanism of BoNT-induced analgesia is not fully understood but may involve modulation of pain pathways and neurogenic inflammation.

Bruxism, characterized by teeth grinding or clenching, is a common contributor to TMDs. BoNT injections have shown promise in reducing bruxism-related muscle activity, thereby preventing further damage to the TMJ.

Additionally, the Herbst appliance can alter the patient’s bite, contributing to TMD symptoms.

It is important to note that not all patients who undergo Herbst treatment will develop TMDs [29, 39]. Factors such as pre-existing TMD symptoms, the severity of the mandibular deficiency, and the length of treatment can all influence the likelihood of developing TMDs. Also, proper diagnosis and management of TMDs can help minimize symptoms and prevent long-term damage to the TMJ [48–50]. An important aid for diagnosis also performed during treatment would be a CBCT [51]. In conclusion, while the Herbst appliance can effectively treat mandibular retrognathism, it can also cause TMDs in some patients [52]. Patients undergoing Herbst treatment should be monitored closely for TMD symptoms, and proper diagnosis and management of TMDs should be implemented if symptoms do arise. Further research is needed to understand better the mechanism by which the Herbst appliance causes TMDs and to develop strategies for minimizing the risk of TMDs in patients undergoing Herbst treatment [53–58].

In addition to the potential for TMDs, other risks are associated with using the Herbst appliance. For example, the device can cause discomfort and difficulty speaking and eating during the initial adjustment period. The Herbst appliance can also cause irritation and sores on the inside of the cheeks and lips. However, these side effects are usually temporary and can be managed with proper care and attention [59].

To minimize the risk of TMDs and other side effects associated with the Herbst appliance, it is important for orthodontists to carefully evaluate each patient before recommending treatment. Patients with a history of TMDs or other jaw problems may not be good candidates for Herbst treatment, and alternative treatment options may be recommended instead [40, 60]. Orthodontists should also closely monitor patients undergoing Herbst treatment for any signs of TMDs or other complications.

Overall, while the Herbst appliance can be an effective treatment option for mandibular retrognathism, patients and orthodontists must be aware of the potential risks associated with the treatment. Proper diagnosis, treatment planning, and monitoring can help to minimize the risk of TMDs and other complications and ensure the best possible outcome for each patient [61, 62].

### Limitations of the study

The meta-analysis has several limitations and strengths. The study analyzes the effects of Herbst therapy on the onset or exacerbation of TMD symptoms. The powers are undoubtedly the homogeneity of the population considering a population with an average age of 12 years. Critical issues of the studies considered, however, are that they do not think other cofactors in the development of TMD and, therefore, bias may be present in the studies. Therefore, more studies will be needed, perhaps with the presence of multiple linear regressions that can evaluate possible confounding factors in the ‘incidence of TMD in patients treated with Herbst.

## Conclusion

Herbst therapy is used in treating Class II malocclusions with increased overate; due to its biomechanical nature, several clinical trials have been conducted regarding the effects of the treatment on TMD situations. The conclusions of the studies in the literature are not in excellent agreement. Therefore, in this meta-analysis, we have analyzed the studies in the literature and have seen no proven statistical evidence on the possible occurrence of TMD after Herbst therapy. However, since other cofactors are not considered in the analyzed studies, our conclusion is weak and needs further studies to be substantiated. In fact, Herbst therapy does not cause an increase in the incidence and prevalence of TMD. However since it still creates muscle tension and anterior condyle slippage, all of which in predisposed individuals, TMDs being of multifactorial etiology, cause an increase in symptoms. Therefore a careful analysis of the joint and muscle profile, as well as psychological, should be carried out on the patient.

## Data Availability

The data will be available on reasonable request from the corresponding author.
